# Diagnostic evaluation of magnetization transfer and diffusion kurtosis imaging for prostate cancer detection in a re-biopsy population

**DOI:** 10.1007/s00330-017-5169-1

**Published:** 2017-12-08

**Authors:** Tristan Barrett, Mary McLean, Andrew N. Priest, Edward M. Lawrence, Andrew J. Patterson, Brendan C. Koo, Ilse Patterson, Anne Y. Warren, Andrew Doble, Vincent J. Gnanapragasam, Christof Kastner, Ferdia A. Gallagher

**Affiliations:** 1 Department of Radiology, University of Cambridge, Cambridge, UK; 2 Department of Radiology, Addenbrooke’s Hospital, Cambridge, CB2 0QQ UK; 3 CamPARI Clinic, Addenbrooke’s Hospital and University of Cambridge, Cambridge, UK; 4 CRUK Cambridge Institute, Cambridge, UK; 5 Present Address: Department of Radiology, Memorial Sloan Kettering Cancer Center, 1275 York Ave, New York, NY USA; 6 Department of Histopathology, Addenbrooke’s Hospital and University of Cambridge, Cambridge, UK; 7 Department of Urology, Addenbrooke’s Hospital and University of Cambridge, Cambridge, UK

**Keywords:** MRI, Prostate, Diffusion kurtosis imaging, Magnetisation transfer imaging, Diffusion-weighted imaging

## Abstract

**Objective:**

To evaluate diffusion kurtosis imaging (DKI) and magnetisation transfer imaging (MTI) compared to standard MRI for prostate cancer assessment in a re-biopsy population.

**Methods:**

Thirty-patients were imaged at 3 T including DKI (K_app_ and D_app_) with b-values 150/450/800/1150/1500 s/mm^2^ and MTI performed with and without MT saturation. Patients underwent transperineal biopsy based on prospectively defined MRI targets. Receiver-operating characteristic (ROC) analyses assessed the parameters and Wilcoxon-signed ranked test assessed relationships between metrics.

**Results:**

Twenty patients had ≥ 1 core positive for cancer in a total of 26 MRI targets (Gleason 3+3 in 8, 3+4 in 12, ≥ 4+3 in 6): 13 peripheral (PZ) and 13 transition zone (TZ). The apparent diffusion coefficient (ADC) and D_app_ were significantly lower and the K_app_ and MT ratio (MTR) significantly higher in tumour versus benign tissue (all *p* ≤ 0.005); ROC values 0.767-1.000. Normal TZ had: lower ADC and D_app_ and higher K_app_ and MTR compared to normal PZ. MTR showed a moderate correlation to K_app_ (*r* = 0.570) and D_app_ (*r* = -0.537) in normal tissue but a poor correlation in tumours. No parameter separated low-grade (Gleason 3+3) from high-grade (≥ 3+4) disease for either PZ (*p* = 0.414-0.825) or TZ (*p* = 0.148-0.825).

**Conclusion:**

ADC, D_app_, K_app_ and MTR all distinguished benign tissue from tumour, but none reliably differentiated low- from high-grade disease.

***Key Points*:**

• *MTR was significantly higher in PZ and TZ tumours versus normal tissue*

• *K*_*app*_ *was significantly lower and D*_*app*_ *higher for PZ and TZ tumours*

• *There was no incremental value for DKI/MTI over mono-exponential ADC parameters*

• *No parameter could consistently differentiate low-grade (Gleason 3+3) from high-grade (≥ 3+4) disease*

• *Divergent MTR/DKI values in TZ tumours suggests they offer different functional information*

**Electronic supplementary material:**

The online version of this article (10.1007/s00330-017-5169-1) contains supplementary material, which is available to authorized users.

## Introduction

Prostate cancer is the second leading cause of cancer in men, accounting for around 20% of male cancer diagnoses [1]. The diagnosis of prostate cancer is primarily based on transrectal ultrasound (TRUS)-guided biopsies. However, this non-targeted sampling approach means around a third of tumours will be under-graded and half missed altogether [2, 3]. In patients with an initial negative biopsy, but continued clinical suspicion of prostate cancer or suspicion of an under-sampled lesion, national guidelines in the UK recommend further assessment to exclude or confirm the presence of aggressive tumour [4]. Multiparametric MRI (mpMRI) is used to guide re-biopsy in such patients and has been shown to outperform systematic TRUS biopsy for lesion detection [5]. However, studies report considerable variation in MRI performance, with sensitivity and specificity ranging from 73-100% and 8-100%, respectively [6, 7], depending on composition of the study population, radiologist experience, technical issues, and the gold standard employed. Furthermore, specificity drops from 80% to 47% when including indeterminate lesions, i.e. Prostate Imaging-Reporting and Data System (PI-RADS) score 3 lesions in addition to score 4-5 [6, 7]. This highlights the need for improvement of existing sequences, or use of additional functional sequences, with the most recent version of the PI-RADS guidelines strongly supporting the continued development of further novel MRI sequences [8]. Here we study two imaging techniques in prostate cancer—diffusion kurtosis imaging (DKI) and magnetisation transfer imaging (MTI). DKI is a novel technique for studying the heterogeneity of water diffusion that has recently been applied to the prostate, and MTI is a more established technique for probing macromolecules in the microenvironment, but there has been limited work using this method in prostate cancer; neither approach requires additional administration of exogenous contrast agents and both could be translated into routine clinical practice if effective.

DKI is a form of diffusion-weighted imaging that quantifies the degree to which water diffusion in tissues differs from what would be expected under a normal (Gaussian) distribution of displacements, and from the corresponding monoexponential decay of signal with increasing b-value [9], deriving two parameters. The apparent diffusivity D_app_ quantifies the exponential component of signal decay and is similar to the apparent diffusion coefficient (ADC) in the standard mono-exponential model. The apparent kurtosis K_app_ measures the first-order deviation from mono-exponential decay and is thus a simple measurement of the deviation from a Gaussian distribution of displacements. Theoretically, kurtosis values may quantify the variability in tissue structure within the region of interest, providing a measurement of intra-voxel tissue heterogeneity, and may be useful for assessing structural abnormalities in pathologic regions. Previous studies have suggested a possible correlation between K_app_ and prostate cancer aggressiveness [10, 11], although a recent large retrospective study showed that despite DKI itself performing well, no added value was observed over standard DWI sequences [12].

MTI detects the interaction between free and bound water molecules. The “free” pool consists of relatively mobile protons and provides the majority of the visible MR signal. The “bound” pool incorporates the “hydration layer” of water molecules bound to the surface of macromolecules. The protons associated with macromolecules are relatively immobile, with decay times being too rapid to detect an MR signal [13], however, the hydration layer is able to interact with the “free” pool and can modulate its relaxation properties and affect the measurable MR signal. This effect is exploited in MTI, where an off-resonance radiofrequency pulse saturates the nuclear magnetisation in the hydration layer; this in turn exchanges with the free pool of protons and thus reduces the MR signal. The MT ratio (MTR) is a simple derived metric and is therefore a measure of the structural integrity of tissues and can probe the microstructural changes induced by pathological processes [14]. MTR has been shown to change in malignancy because of changes in cell number and size as well as changes in cell membrane structures and extracellular space content, which may help differentiate primary for secondary brain tumours [15, 16]. Preliminary work in prostate cancer has demonstrated a higher MTR within the peripheral zone of patients with prostate cancer compared to normal controls [17, 18].

Advanced functional imaging with DKI and MTI may provide insight into the tissue structure and the complicated micro-environment of prostate tumours. We therefore aimed to evaluate whether the addition of these two novel MR quantification parameters to standard MRI sequences could aid prostate cancer detection in a transperineal re-biopsy population.

## Methods

### Patient population

Thirty-patients with a clinical suspicion of undiagnosed prostate cancer were prospectively enrolled into this local institutional review board-approved (CUH/13/EE/0100) single-centre study between November 2013 and June 2016, with all subjects signing written informed consent. Inclusion criteria included prior negative biopsy with a suspicious lesion on MRI (*n* = 24) or prior diagnosis of low-grade prostate cancer and an MRI suspicious lesion in a remote gland location (*n* = 6). Patients subsequently underwent MR-TRUS fusion template transperineal biopsy, including target cores from MRI suspicious lesion/s.

### MR Imaging

All patients underwent 3-T MRI (MR 750, GE-Healthcare, WI, USA) using a 32-channel phased-array coil. The protocol included multiplanar T2-weighted fast recovery fast spin-echo (FSE) images of the prostate and axial T1-weighted FSE images of the pelvis. Standard clinical axial DWI was performed using a single spin-echo echo-planar imaging (EPI) pulse sequence with b-values of 150/1000/1400 s/mm^2^, with automated ADC maps. DCE was performed as a 3D fast-spoiled gradient echo sequence; 85–100 dynamic phases were acquired with temporal resolution 7 s; bolus gadobutrol (Schering AG) was injected intravenously via a power injector (rate 3 ml/s, dose 0.1 mmol/kg) followed by 25 ml saline flush; total scan duration was 10 min.

DKI was performed as a single-shot dual-spin-echo EPI pulse sequence, including five different b-values and a ‘noise-only’ image set with no RF pulses but otherwise identical acquisition parameters. MTI volumes covering the prostate were acquired with two 3D spoiled gradient recalled-echo acquisitions with and without an MT saturation pulse with slice thickness matched to T2-axial images. The saturation pulse consisted of a 400° Fermi shape pulse of 10-ms duration and 800 Hz bandwidth at 2.5 kHz off-resonance frequency (Table 1).

**Table 1.** Sequences in MRI protocol

Parameter	Axial T2 2D FSE	DWI	DKI	MTI
TR (ms)	3000-5000	4000	6000	24
TE (ms)	99-106	70–75	94	2.4 / 4.8
Averages	3	8	6	1
Section thickness (mm)	3	4	3.6	4
Section gap (mm)	1	0	0.4	0
FOV (mm)	220 × 220	280 × 280	280 × 280	220 × 220
Matrix	384 × 288	128 × 128	128 × 96	192 × 160
Resolution (mm^2^)	0.6 × 0.8	2.2 × 2.2	2.2 × 2.9	1.15 × 1.38
Receiver bandwidth (± kHz)	50	111	111	31
ASSET factor	No ASSET	2	2	No ASSET
Time	4:39	2:58	11:30	2:50
Other	ETL 16 No phase wrap	b-values 0, 1400 s/mm^2^	b-values 150, 450, 800, 1150, 1500 s/mm^2^	Flip angle 5°

FSE, fast spin echo; EPI, echo planar imaging; DWI, diffusion-weighted imaging; FOV, field of view; ETL, echo train length; DKI, diffusion kurtosis imaging; MTI, magnetisation transfer imaging

### MRI-Guided Biopsy

The MRIs were prospectively interpreted by one of two uroradiologists with > 4 years’ prostate MRI reporting experience. Images were analysed according to PI-RADS version 1 criteria [19] prior to February 2015 (*n* = 13) and subsequently using criteria described in PI-RADS version 2 (*n* = 17) [4]. In all cases, interpretation was based on a Likert scale: 1, no suspicious area; 2, cancer unlikely; 3, indeterminate; 4, cancer likely; 5, cancer highly likely [19–22]. All studies were reviewed in a multidisciplinary team environment, which included radiologists, urologists, and oncologists, with all the clinical information available prior to the decision to undertake a biopsy. A positive lesion was defined as a Likert score ≥ 3. DKI and MTR values did not inform biopsy decision-making. The Biopsee™ MRI/TRUS-fusion biopsy system v.1 or v.2 (Medcom, Darmstadt, Germany) was used for all biopsies. A transrectal FlexFocus™ (BK-Medical, MA, USA) ultrasound probe was sited; the biplanar probe is fixed on a stepping unit and sagittal prostate images aligned with MRI using fusion software. Targets were prospectively drawn using T2W as primary and ADC as secondary source images. All patients underwent systematic transperineal biopsies according to the Ginsburg protocol, using a spring-loaded 18-G biopsy needle via a brachytherapy template grid [23]. In all cases two biopsy cores were taken from each lesion(s) first, with 24 background systematic biopsies subsequently acquired. All procedures were performed by one of three urologists with several years’ experience of transperineal biopsy. All biopsies were Gleason-graded by a specialist uropathologist, following ISUP 2005 recommendations [24].

### Image Analysis

Prospectively defined target outlines are stored on the local PACS system during the clinical workflow. A fellowship-trained uroradiologist with 7-years’ clinical prostate MR reporting experience reviewed these original outlines and re-drew freehand ROIs on T2-weighted axial images, avoiding inclusion of the urethra or extra-prostatic tissue where relevant, using in-house software programmed with MATLAB (version 2016a; MathWorks, Natick, MA, USA). Additional ROIs were drawn in regions of the biopsy-benign peripheral zone (PZ) and transition zone (TZ) from the side contralateral to the target over three consecutive slices, with a minimum volume of 0.5 cm^3^. ROIs were secondarily transposed onto ADC, DKI, and MTR maps; in cases with significant distortion on any of the sequences, ROIs were adjusted to allow for this, using the targets and prostate outline on T2-weighted images for reference. DKI (D_app_ and K_app_) and MTR parameter maps were calculated offline using custom software written in Matlab. For DKI, the noise-only images were used to reduce and partially compensate for noise-floor bias, which could otherwise artificially enhance the kurtosis measurement [10]. A small proportion of DKI fit-failure pixels were excluded. The mean ROI values of ADC, D_app_, and K_app_ and MTR maps were used for analysis.

### Statistical analysis

Medians and ranges were used to summarise continuous variables. Wilcoxon signed-rank test assessed the relationships between parameters, benign versus tumour tissue, and tumour grades in both the PZ and TZ. Receiver-operating characteristic (ROC) analyses were used to assess the diagnostic utility of metrics for detecting tumour and for discriminating Gleason grade. An optimal threshold was determined for each metric for discriminating tumour from benign tissue. Pearson’s correlation assessed the relationship between the metrics. All statistical analysis was performed in R (version 3.1.1, The R-Foundation, Austria); *p* < 0.05 was considered statistically significant.

## Results

Thirty patients were included, with a median age of 65.5 years (range 50–76 years) and a median PSA of 7.67 ng/ml (IQR: 6.12-11.98 ng/ml). The median time from MRI to biopsy was 26 days (IQR: 7.25–48.25 days). Twenty-six patients had a least one previous biopsy, with the interval from the most recent biopsy to MRI being at least 3 months (median 13, range 3-114, IQR 4–26 months).

**Fig. 1.**
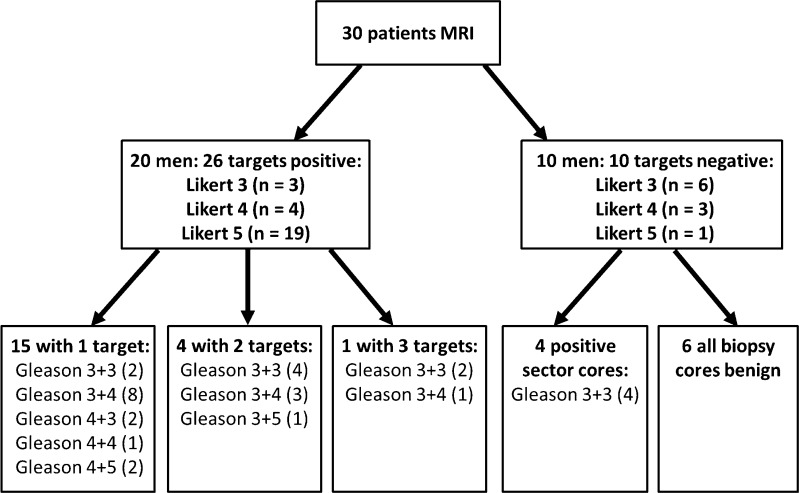
Flow chart of biopsy outcomes

Prostate cancer was detected in 24/30 patients including targeted and background cores and in 20 patients within at least one target core. In the four patients with only positive background cores, all had a Gleason score (GS) 3+3 in 1-5% of one core only. The remaining 20 patients had 26 separate MRI lesions with positive target cores (4 patients with 2 targets, 1 with 3), of which 13 were in the peripheral zone (PZ) and 13 in the transition zone (TZ) (Fig. 1). The final pathology of the 26 targets was GS 3+3 (*n* = 8), 3+4 (12), 3+5 (1), 4+3 (2), and ≥ 4+4 (3) (supplemental Table 1). The mean size for these 26 targets was 1.16 cm^2^ (range 0.22 – 4.71 cm^2^).

Mean ADC and D_app_ values for tumours in both the PZ and TZ were significantly lower than comparative normal PZ and TZ tissue (all *P*-values < 0.001) (Table 2). Conversely, mean K_app_ and MTR were significantly higher for PZ tumour and TZ tumour compared to normal PZ and normal TZ (*P* ≤ 0.005). Normal TZ had lower ADC and D_app_ and higher K_app_ and MTR than normal PZ tissue, with cystic BPH demonstrating higher ADC and D_app_ and lower K_app_ and MTR compared to normal TZ (all *P*-values ≤ 0.005). For all measured parameters, there was a significant difference between normal tissue and tumour and between cystic BPH and normal TZ (all *P* ≤ 0.005).

**Table 2.** Target biopsy outcomes for MRI-defined lesions and contralateral benign tissue

Parameter	Normal tissue	Tumour target	Cystic BPH	*p*-value(normal vs. tumour)	*p*-value(normal vs. cystic BPH)
*ADC* (× 10^-3^ mm^2^s^-1^)					
Peripheral zone	1.579 [1.363 – 1.794]	0.902 [0.869 – 0.974]	N/A	< 0.001*	N/A
Transition zone	1.270 [1.244 – 1.355]	0.845 [0.813 – 0.885]	1.955 [1.669 – 2.031]	< 0.001*	< 0.001
*D*_*app*_ (× 10^-3^ mm^2^s^-1^)					
Peripheral zone	2.221 [2.039 – 2.446]	1.167 [0.910 – 1.366]	N/A	< 0.001*	N/A
Transition zone	1.808 [1.712 – 1.895]	0.951 [0.853 – 1.062]	2.152 [2.113 – 2.258]	< 0.001*	< 0.001*
*K*_*app*_ *(unitless)*					
Peripheral zone	0.507 [0.426 – 0.550]	0.716 [0.621 – 0.869]	N/A	0.004*	N/A
Transition zone	0.615 [0.548 – 0.653]	0.871 [0.663 – 0.1034]	0.374 [0.303 – 0.501]	0.004*	0.001*
*MTR (%)*					
Peripheral zone	20.0 [15.5 – 22.9]	25.0 [24.0 – 28.1]	N/A	< 0.001*	N/A
Transition zone	26.9 [23.5 - 28.0]	30.0 [28.2 - 32.0]	15.2 [6.9 - 21.3]	0.005*	< 0.001*

Mean values listed, interquartile range in parentheses; *p < 0.05. BPH, benign prostatic hyperplasia

**Fig. 2.**
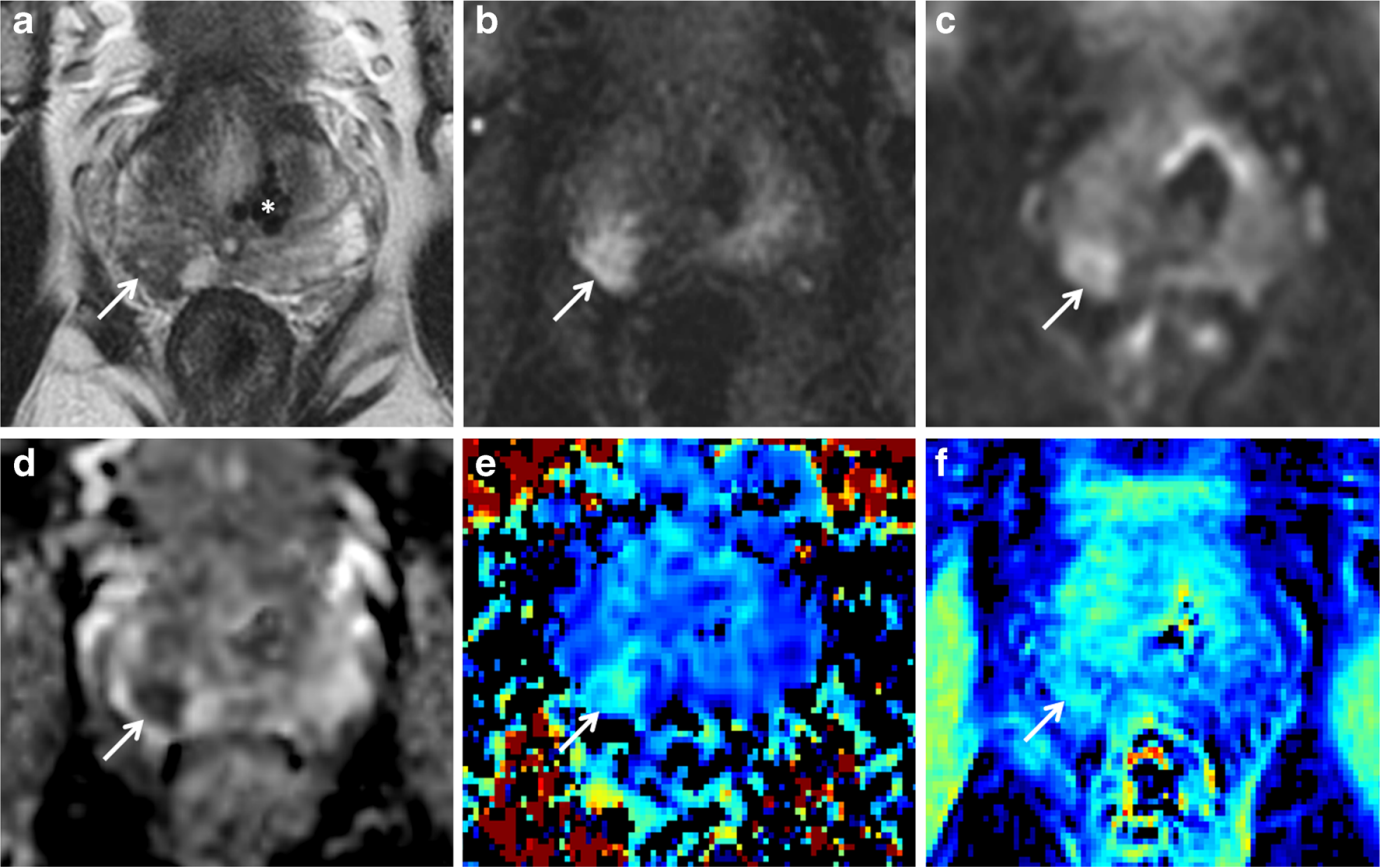
Peripheral zone target. A 64-year-old male with a PSA of 6.5 ng/ml. Top row: clinical mpMRI sequences: T2w image (**a**), early time point DCE image (**b**), and b = 1400 s/mm^2^ diffusion-weighted image (**c**). Bottom row: assessed sequences: ADC map (**d**), K_app_ (**e**), and MTR (**f**). Target prospectively drawn in the right base PZ posteriorly (arrows). Note calcification in the left base TZ (* in **a**) with corresponding artefact on all other sequences. Target biopsy: 3 + 4 = 7 (35% pattern 4) in 60% cores

**Fig. 3.**
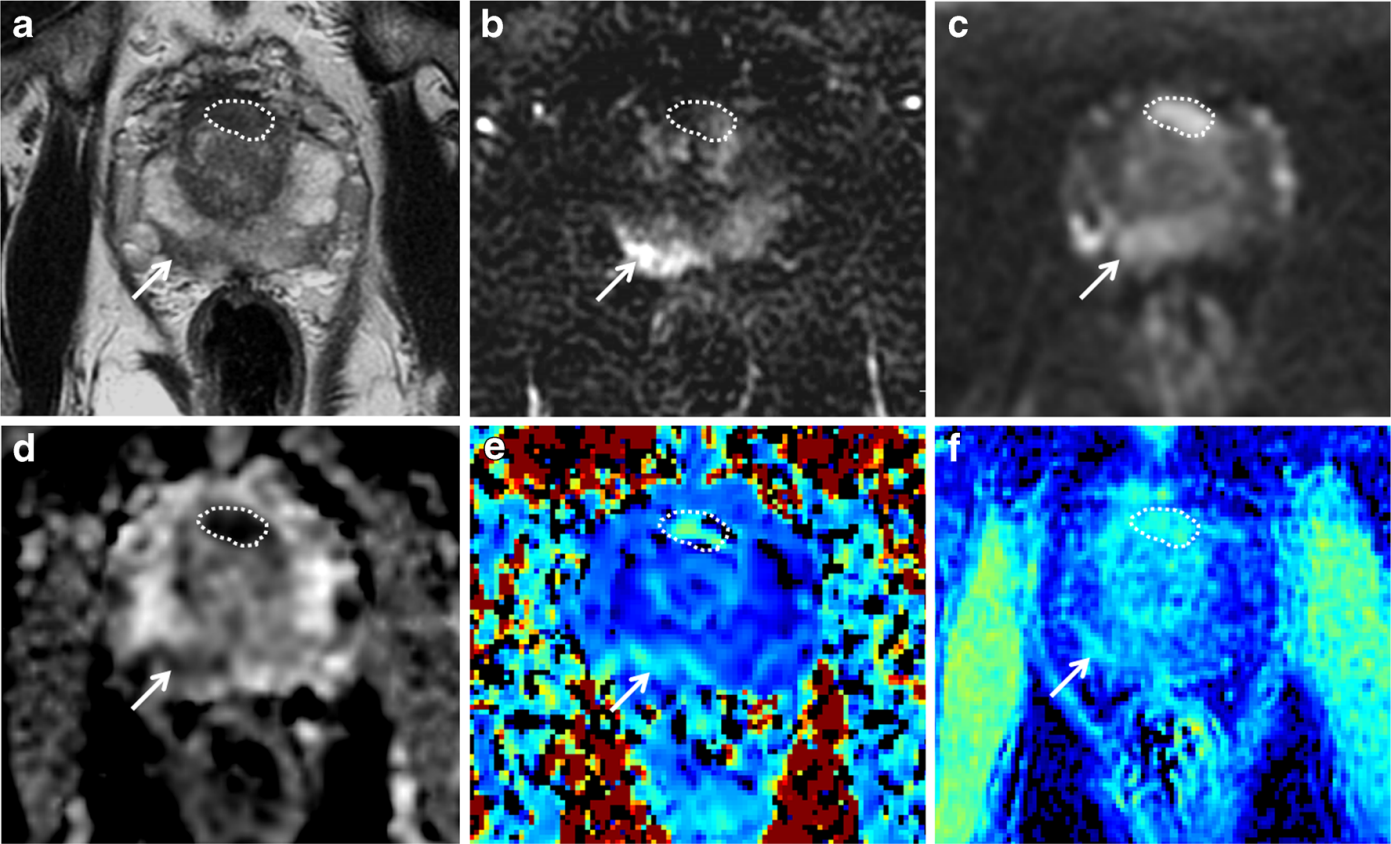
Peripheral and transition zone target. A 69-year-old male with PSA of 12.31 ng/ml. Top row: clinical mpMRI sequences: T2w image (**a**), early time point DCE image (**b**), and b = 1400 s/mm^2^ diffusion-weighted image (**c**). Bottom row: assessed sequences: ADC map (**d**), K_app_ (**e**), and MTR (**f**). Targets prospectively drawn in the anterior mid gland TZ (outlines) and right mid PZ posteriorly (arrows). Both lesions positive on K_app_ and MTR maps; note clear zonal differentiation seen on MTR maps. Target biopsy, PZ lesion: Gleason 3+4 in 2/2 cores 50%, up to 8 mm; TZ lesion: Gleason 3+3 in 2/2 cores 50%, up to 4 mm

**Fig. 4.**
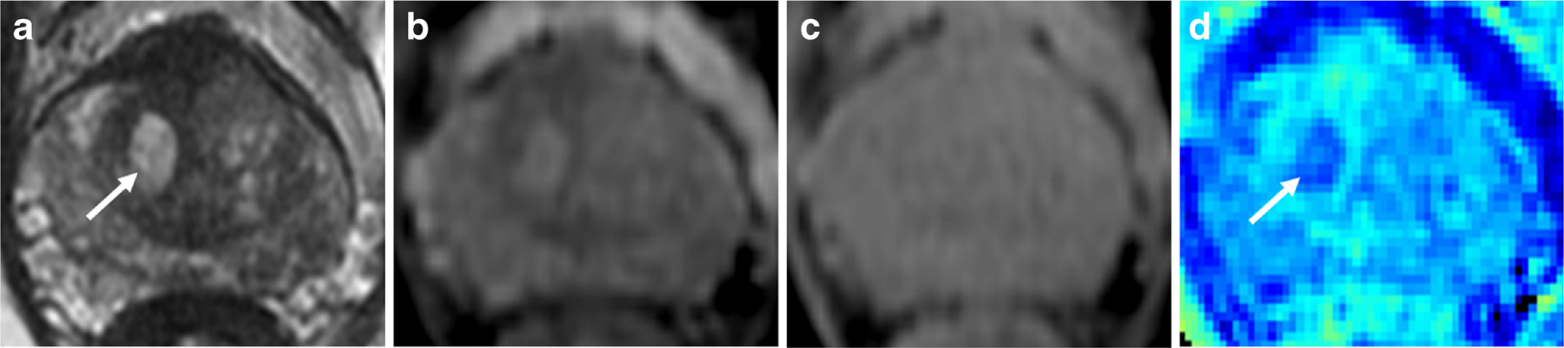
Cystic BPH demonstrated by magnetisation transfer imaging. A 67-year-old patient, PSA 6.39 ng/ml; target biopsy shows Gleason 3+4 tumour in the left mid TZ (not shown). T2-weighted imaging (**a**) shows a cystic area of BPH in the right apex transition zone (arrow); the area appears as high signal with MT “on” imaging (**b**), isointense on MT “off” imaging (**c**), and low signal on the magnetization transfer ratio image (**d**)

**Fig. 5.**
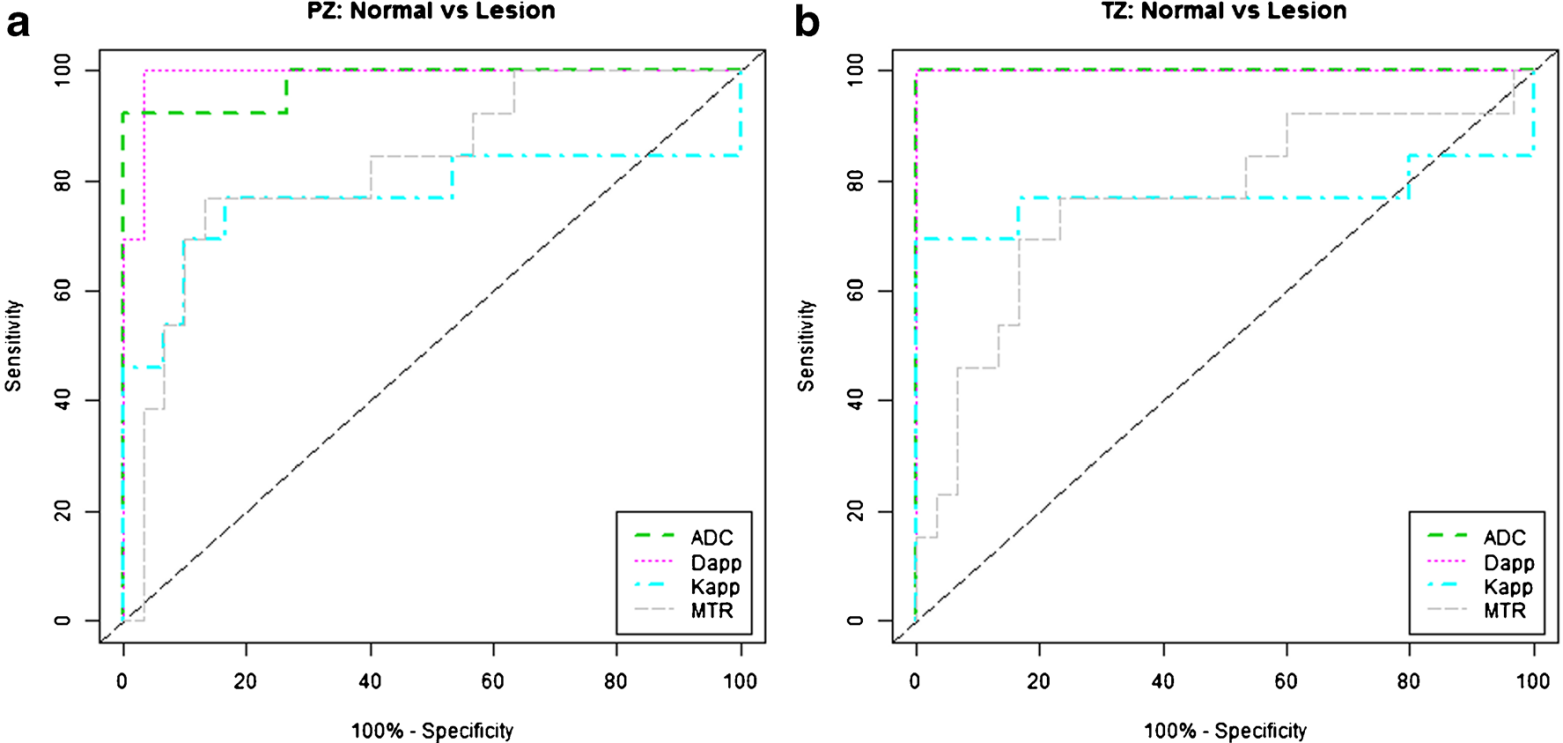
Receiver-operating characteristic curves of the performance of ADC, D_app_, K_app_, and MTR for differentiating benign tissue and tumour in the peripheral (**a**) and transition zone (**b**)

ADC was able to distinguish tumour from benign tissue with a sensitivity and specificity of 92.3% and 100% in the PZ and 100% and 100% in the TZ, respectively. K_app_ differentiated tumour in the PZ with 76.9% sensitivity and 83.3% specificity and in the TZ with 69.2% sensitivity and 100% specificity. MTR distinguished tumour from benign tissue with sensitivity and specificity of 76.9% and 86.7%, respectively, in the PZ and 76.9% and 76.7%, respectively, in the TZ (Figs. 2, 3 and 4). The ROC area-under-the-curve (AUC) values from the diagnostic metrics ranged from 0.767 to 1.000 for separating tumour from benign tissue (Table 3, Fig. 5). A comparison of AUCs using the bootstrapping method showed no statistically significant difference between ADC and D_app_ in the TZ or PZ (*p* = 1.0 and 0.670, respectively). However, the diagnostic utility measured using AUCs was significantly better for ADC compared to K_app_ and MTR in both the TZ (*p* = 0.036 and 0.007) and PZ (*p* = 0.028 and 0.014).

**Table 3.** Diagnostic utility of each respective metric in separating normal tissue vs. tumour

	Cut-off	AUC	Sensitivity (%)	Specificity (%)
*ADC* × 10^-3^ mm^2^s^-1^				
Transition zone	1.076	1.000	100.0	100.0
Peripheral zone	1.037	0.979	92.3	100.0
*D*_*app*_ × 10^-3^ mm^2^s^-1^				
Transition zone	1.524	1.000	100.0	100.0
Peripheral zone	1.481	0.990	100.0	96.7
*K*_*app*_ (unitless)				
Transition zone	0.820	0.772	69.2	100.0
Peripheral zone	0.621	0.772	76.9	83.3
*MTR (%)*				
Transition zone	28.2	0.767	76.9	76.7
Peripheral zone	24.0	0.828	76.9	86.7

AUC, area under the curve

The ability of the parameters to distinguish low- (Gleason 3+3) from high-grade (Gleason ≥ 3+4) was also assessed. A separate analysis was performed for PZ and TZ because of the different values derived for normal tissue for all metrics. The Gleason 3+3 group included four PZ and four TZ tumours; the GS ≥ 3+4 included nine PZ and nine TZ tumours. All measured parameters were poor at separating low- and high-grade disease for both PZ (*p* = 0.414-0.825) and TZ (*p* = 0.148-0.825) (Table 4).

**Table 4.** Ability of metrics to distinguish low- (Gleason 3+3) from high-grade (Gleason ≥ 3+4) tumours

	Gleason 3+3	Gleason ≥ 3+4	*p*-value
Peripheral zone	*n = 4*	*n = 9*	
ADC × 10^-3^ mm^2^s^-1^	0.905 [0.860 – 0.949]	0.902 [0.893 – 0.1036]	0.711
*D*_*app*_ × 10^-3^ mm^2^s^-1^	1.275 [1.047 – 1.463]	1.167 [0.740 – 1.196]	0.414
*K*_*app*_ (unitless)	0.759 [0.643 – 0.902]	0.716 [0.480 – 0.867]	0.711
MTR (%)	26.1 [23.4 – 28.4]	25.0 [24.0 – 27.5]	0.825
*Transition zone*	*n = 4*	*n = 9*	
ADC × 10^-3^ mm^2^s^-1^	0.881 [0.864 – 0.887]	0.830 [0.799 – 0.884]	0.414
D_app_ × 10^-3^ mm^2^s^-1^	0.926 [0.889 – 0.979]	1.006 [0.745 – 1.083]	0.825
K_app_ (unitless)	0.695 [0.508 – 0.924]	0.975 [0.820 – 1.035]	0.414
MTR (%)	31.9 [31.3 – 32.2]	29.6 [26.3 – 31.5]	0.148

Mean values listed, interquartile range in parentheses

ADC showed a strong overall correlation to D_app_ (*r* = -0.862), as expected. ADC showed a moderate inverse correlation to MTR (*r* = -0.618) and a good inverse correlation to K_app_ (*r* = -0.767) in normal tissue, with a lower correlation in tumour tissue (*r* = -0.459 and -0.444, respectively) (Table 5). MTR showed a moderate correlation to both K_app_ (*r* = 0.570) and D_app_ (*r* = -0.537) in normal tissue, but conversely showed a notably poor correlation to K_app_ (*r* = 0.141) and D_app_ (*r* = -0.024) in tumour tissue.

**Table 5.** Correlation between assessed metrics (Pearson’s Rho)

Comparators	Tumour Tissue (TZ + PZ)	Normal Tissue (TZ + PZ)	Normal + Tumour Tissue (TZ + PZ)
**ADC vs K**_**app**_	**-0.444**	**-0.767**	**-0.641**
ADC vs D_app_	-0.041	-0.790	0.862
**ADC vs MTR**	**-0.459**	**-0.618**	**-0.633**
K_app_ vs D_app_	0.646	-0.532	-0.359
**K**_**app**_ **vs MTR**	**0.141**	**0.570**	**0.429**
D_app_ vs MTR	-0.024	-0.537	-0.531

## Discussion

In this study we assessed the potential added value of magnetisation transfer imaging and non-Gaussian diffusion kurtosis imaging to conventional mpMRI sequences for the detection of prostate cancer in a re-biopsy population, using targeted transperineal biopsy as the reference standard. All four measured parameters were able to distinguish benign from tumour tissue, but performed poorly at differentiating low- (GS 3+3) from high-grade (≥ 3+4) disease. Standard diffusion-weighted imaging ADC maps showed a moderate overall inverse correlation with both K_app_ and MTR, but there was no observable correlation between MTR maps and DKI parameters within tumours.

A number of previous studies have looked at the ability of DKI to differentiate tumour grade compared to standard diffusion-weighted imaging with mono-exponential modelling. DKI parameters have repeatedly been shown to distinguish benign from tumour tissue, with several studies suggesting that the kurtosis metric K outperforms ADC for differentiating low- and high-grade tumours [10, 11, 25–27]. However, these studies also note a strong inverse correlation between K with ADC, as supported by our data, raising the question of the additional clinical benefit over existing DWI sequences, given the increased technical complexity of DKI in terms of post-processing and interpretation. Indeed, more recent work and a large retrospective study using prostatectomy as a reference standard suggested no additional benefit of DKI sequences over conventional DWI [12, 28]. A possible explanation is the use of a clinically derived ADC map in these studies (using a high b-value of 1000) in contrast to earlier work where the ADC map was derived from the DKI sequences with high b-values ranging from 1400-2000 s/mm^2^. Current guidelines caution against using such high b-values for ADC calculation because of the non-mono-exponential decay and concerns about a reduced signal-to-noise ratio and noise-floor bias [8]; this may therefore have affected the performance of the ADC measurements in these studies. We also derived ADC maps from standard clinical DWI sequences, which may explain the lack of added benefit of DKI parameters. Another explanation may be the longer echo time (TE) used in the DKI sequence to achieve the diffusion weighting, which reduced the intrinsic SNR of the D_app_ obtained using lower b-values, as suggested by Roethke et al. [28].

Two previous studies have investigated the utility of MTI in prostate cancer [17, 18]. Both were performed at 1.5 T and assessed only PZ tumours, used systematic TRUS biopsy as the reference standard, and did not differentiate between Gleason grades. Our results support the findings of these studies that normal TZ and PZ tumours have a higher MTR than normal PZ. The difference in MTR between normal PZ and normal TZ is expected given the difference in composition between the tissue types: the lower value in the PZ may be explained by a relatively loose stroma, a larger extracellular space, and a fluid-filled glandular cavity [29]. The mean MTR values derived here are comparable to those in the study by Arima et al. [18] for both normal TZ (26.9% vs. 25.5%) and PZ tumour (25.0% vs. 30.6%), but higher than the study by Kumar et al. (7.01% and 8.29%, respectively) [17]. Conversely our results for normal PZ (20.0%) were much higher than these two studies (8.0% and 6.15%, respectively). This discrepancy may relate to the fact that MTR is an arbitrary measure and depends on the characteristics of the pulse sequence; the Kumar et al. study differed from ours in being a 2D sequence with a long TR. In addition, the earlier studies may have effects relating to residual post-biopsy haemorrhage, which will predominantly affect the PZ rather than the TZ when systematic TRUS biopsy is performed, but will typically be excluded from areas containing cancer [30]; thus only normal PZ rather than TZ or PZ tumour will be affected.

A number of previous studies have shown ADC to decrease as tumour Gleason grade increases, albeit with a degree of overlap in values between tumour grades [31–33]. Although there was a trend for lower ADC values in higher-grade (Gleason ≥ 3+4) tumours in our cohort, this was non-significant, which may relate to the relatively small sample size, particularly within the PZ. However, the concordance between ADC and Gleason grade has recently been questioned [34], with the demonstration that Gleason 3+3 tumours can have low ADC values [35]. This highlights the need for further work in the area to both improve the understanding of ADC metrics in this regard and assess additional better functional sequences for characterisation of tumour aggressiveness. MTR showed a moderately strong correlation to both K_app_ (*r* = 0.57) and D_app_ (*r* = -0.537) in normal tissue, yet no correlation in tumour tissue (*r* = 0.141 and -0.024, respectively). Of note, there was a non-significant trend for increasing K_app_, but conversely lower MTR in higher-grade transition zone tumours. These divergent results combined with the poor correlation of MTI and DKI suggest that the techniques offer different but complementary information on the tumour microenvironment. Interestingly, both tumour K_app_ and MTR showed a high inverse correlation to ADC (*r* = -0.444 and -0.459, respectively), suggesting that cellularity is partly contributing to these metrics, whilst other factors are contributing to their divergent values. At higher Gleason grades, the glandular structure of the prostate is progressively disrupted with increased cellularity and a reduction in the stromal matrix and luminal space [21]. This increased heterogeneity can therefore help to explain a trend for higher K_app_ values observed in higher-grade tumours [10]. Higher-grade prostate cancer is expected to result in an increased number of intracellular bound water molecules due to increased cellular density [36]; however, this is counteracted by a breakdown of the normal extensive extracellular matrix [37]. These competing processes may provide an explanation for the divergence among DWI, DKI, and MTI demonstrated here in tumours.

Our study has a number of limitations. The numbers within the cohort were relatively low; this may have particularly affected attempts to differentiate high- and low-grade tumours, where further sub-division became necessary. Targeted biopsy ensured that tissue was sampled from the outlined lesion; however, as with any biopsy technique, this is prone to sampling error and may misclassify the grade compared to the more robust gold standard of prostatectomy [38]. Targets were prospectively chosen based on clinical mpMRI sequences, and DKI/MTR values were not used to inform the biopsy decision. This may have led to a bias towards lesions with restricted diffusion and low ADC, particularly within the PZ where this is considered the key diagnostic sequence. Conversely, there may be a bias against areas that demonstrated changes on DKI or MTR but not on conventional MRI sequences as they would not have been biopsied.

In conclusion, ADC, MTI, and DKI readily distinguished benign tissue from tumour, but none of the measured parameters reliably differentiated low- from high-grade disease. Differences between DKI and MTI at higher Gleason grades may be explained by changes in the cellularity, stromal matrix, and luminal space. DKI and MTI may therefore offer different but potentially complementary information on the tumour microenvironment.

## Electronic supplementary material

Supplemental Table 1 (DOCX 15 kb)
