# Supplementary material for: Diagnostic evaluation of magnetization transfer and diffusion kurtosis imaging for prostate cancer detection in a re-biopsy population
Source: Eur Radiol. 2017 Dec 8;28(8):3141–50. doi: 10.1007/s00330-017-5169-1 (PMC6028858; doi:10.1007/s00330-017-5169-1)
Supplement: Supplementary file 1 — (DOCX 15 kb) [file 330_2017_5169_MOESM1_ESM.docx]

**Supplemental Table 1. Target biopsy outcomes for MRI defined lesions**

| **Gleason Grade** | **Overall** | **Peripheral Zone** | **Transition Zone** |
| --- | --- | --- | --- |
| Benign | 10 | 3 | 7 |
| 3+3 | 8 | 4 | 4 |
| 3+4 | 12 | 8 | 4 |
| 3+5 | 1 | 0 | 1 |
| 4+3 | 2 | 0 | 2 |
| 4+4 | 1 | 1 | 0 |
| 4+5 | 2 | 0 | 2 |
